# Si_2_Ge: A New VII-Type Clathrate with Ultralow Thermal Conductivity and High Thermoelectric Property

**DOI:** 10.1038/s41598-020-59820-8

**Published:** 2020-02-20

**Authors:** Jinni Shen, Tianzhu Xie, Longkun Zhang, Ping Wang, Zhenxing Fang

**Affiliations:** 1 College of Materials Science and Engineering, Fuzhou University, Fuzhou, 350108 China; 2 Key Laboratory of Eco-materials Advanced Technology, College of Materials Science and Engineering, Fuzhou University, Fuzhou, 350108 China; 3 Department of Physics, Zunyi Normal University, Zunyi, Guizhou 563006 China

**Keywords:** Energy, Electronic structure

## Abstract

Based on global particle-swarm optimization algorithm and density functional theory methods, we predicted an alloyed Si_2_Ge compond with body centered tetragonal type VII clathrate (space group *I*4/mmm) built by a truncated octahedron fromed by six quadrangles and eight hexagons ([4^6^6^8^]). Si_2_Ge clathrate is 0.06 eV/atom lower than VII Si clathrate and thermally stable up to 1000 K. It has an indirect band gap of 0.23 eV, high p-doping Seebeck coefficient and n-doping electrical conductivity. It owns a low lattice thermal conductivity of 0.28 W/mK at 300 K because of its weak bonding and strong anharmonic interaction of longitudinal acoustic and low-lying optical phonons. The moderate electronic transport properties together with low lattice thermal conductivity results in a high optimal thermoeletric performance value of 2.54 (1.49) at 800 (1000) K in n (p)-doped Si_2_Ge.

## Introduction

Thermoelectric (TE) materials can realize the mutual conversion between heat and electricity, the search and preparation of high-performance TE materials have been received a great deal of attention from environment and energy communities. The efficiency is generally expressed as a dimensionless TE figure-of-merit (ZT). High ZT value depends on high Seebeck coefficient (*S*), high electronic conductivity (*σ*) and low thermal conductivity (*κ*) including electronic (*κ*_e_) and lattice contributions (*κ*_L_). These parameters are coupled with each other, so it is difficult to regulate independently and then improve TE performance. The ideal TE materials possess the structure only suppresses the movement of phonon and not the electrons^1^. These materials are called as phonon glass electron crystal, or PGEC for short^2,3^. Clathrates are one of PGEC materials and are considered as a newly classes of potential TE materials^3,4^.

Inorganic clathrates are those “open-structured” compounds consisting of 3D network framework mostly formed by group 14 atoms (Si, Ge or Sn) through covalent tetrahedral bonds, creating cavities or cages in which metal atoms are embedded^5,6^. The clathrates have been gain more interesting because of their transport properties and wide band-gap range show promising TE^4,7,8^ and optical application^9^. Although the clathrates have so many advantages, their ZT is below 0.2 for Si-based due to their poor power factor^10–12^. The Seebeck coefficient and lattice thermal conductivity of the Ge and Sn-based clathrates are superior to those of the Si-based, resulting in a larger ZT^13–15^. Additionally, SiGe alloyed clathrates exhibit a significant increase in TE performance from their high power factor (PF, *S*^2^*σ*) and low lattice thermal conductivity^16^. Apart from this, SiGe alloyed clathrate have a great potential in terms of superior optical^17^ and electrical properties^18^. This phenomenon does not only exist in the clathrates, many researches reveal that alloyed inducing band convergence is responsible for the high Seebeck coefficients^19,20^ and tuning the electrical properties as result of modifying the band structure^16^, meanwhile increasing the phonon scattering in order to reduce the thermal conductivity^20,21^, and thus leading to a significant increase of TE performance in alloy compounds^22,23^. Therefore, a clathrate containing both Si and Ge atoms with moderate electron and phonon transport will be of great TE performance.

Herein we report a new Si_2_Ge-clathrate compound with the sodalite-type structure using global particle-swarm optimization algorithm and density functional theory. This clathrate is made up of a (Si_8_Ge_4_)2 tetrakaidecahedra, which could be extending in a 2 × 2 × 2 supercell with a structure like conventional sodalite. It has a 0.23 eV indirect band gap. Such a Si_2_Ge clathrate is 0.06 eV/atom lower than the Si-VII type clathrate, and holding the cage configuration up to 1000 K. Si_2_Ge is essentially guest-free and possesses a very low thermal conductivity of 0.28 W/mK at 300 K because of the strong coupling between longitudinal acoustic (LA) and low-lying optical (LLO) phonons. This coupling reveals an avoided-crossing behaviour of LA and LLO originates from an anharmonic interaction. Furthermore, the calculated Seebeck coefficient and electronic conductivity suggest desirable TE properties in this Si_2_Ge clathrate. The optimized ZT value is about 2.54 and 1.49 for n and p-doping Si_2_Ge clathrate.

## Methods

### Structure prediction

We employ the efficient particle swarm optimization (CALYPSO) code^24^ to search for low-energy 3D Si_2_Ge clathrate. The number of formula units per simulation cell is set to be 1~2. Unit cells containing total number atoms of 6 and 12 are considered. The structure relaxations are performed using Vienna ab initio simulation package (VASP)^25,26^. The projector-augmented plane wave (PAW) approach^27^ is used to represent the ion-electron interaction. The generalized gradient approximation in the form of Perdew, Burke and Ernzerhof (PBE) is adopted^28^. The plane-wave cutoff energy for wave function is set to 600 eV. Monkhorst-Pack k-mesh of 5 × 5 × 5 is adopted to represent the first Brillouin zone. For structure optimization, the convergence thresholds are set to 10^−7^ eV and 10^−3^ eV/Å for total energy and force component, respectively.

### Electronic and phonon structure

The Heyd-Scuseria-Ernzerhof (HSE06) hybrid functional^29,30^ are also used for the high accuracy of electronic structure calculations. The plane-wave cutoff energy for wave function is set to 400 eV. Monkhorst-Pack k-mesh of 7 × 7 × 7 is adopted to represent the first Brillouin zone. Ab initio molecular dynamics (AIMD) simulations at different temperatures are performed using the canonical ensemble (NVT) with the Nosé thermostat^31^ to examine thermal stability. Simulations lasted for 10 ps with a time step of 1 fs at the temperature of 500, 1000, and 1200 K were carried out. Phonon spectrum calculation is carried out using the linear response method within density functional perturbation theory^32^ implemented in the Phonopy code^33^.

### TE performance calculation

Based on the Boltzmann transport theory, the Seebeck coefficient, the ratio of electrical conductivity to electrical relaxation time and the electronic thermal conductivity are evaluated by using the semiclassical Boltzmann transport theory with the relaxation time approximation, which is implemented in the so-called BoltzTraP code^34^. Here it is assumed that the acoustic phonon is the main scattering mechanism, we calculated carrier mobility by the deformation potential (DP) theory^35^ as following^36,37^

1 $$\mu =\frac{\tau e}{{m}_{{\rm{{\rm I}}}}^{\ast }}=\frac{{2}^{\frac{3}{2}}{\pi }^{\frac{1}{2}}{\hslash }^{4}\rho {\upsilon }^{2}e}{3{m}_{{\rm{{\rm I}}}}^{\ast }{({m}_{s}{k}_{{\rm{{\rm B}}}}{\rm{{\rm T}}})}^{3/2}{E}_{1}^{2}}$$

where *μ* is carrier mobility, ${m}_{{\rm{{\rm I}}}}^{\ast }$ is inertial effective mass, *m*_s_ is the density of states effective mass of a single band, *ρ* is the crystal mass density, *υ* is the average sound velocity from phonon dispersion listed in Table S1 (Supplementary Information). The term *E*_1_ represents the deformation potential constant of the valence-band minimum (VBM) for hole or conduction-band maximum (CBM) for electron along the transport direction. The deformation potential constant (*E*_1_) is calculated by the linear fitting of the CBM (VBM)–strain relation, the result is shown in Fig. S1 (Supplementary Information). With *E*_1_, and the effective mass is known, the carrier motilities are calculated by Eq. (1).

### Lattice thermal conductivity

The first-principles lattice thermal conductivity *κ*_L_ was calculated by solving Boltzmann transport equation for phonons. The interatomic force constants (IFCs) were calculated within a real-space supercell approach using the Phonopy package^33^ for the two-order harmonic IFCs and the ShengBTE package^38^ for the thirdorder anharmonic IFCs. The IFCs were calculated using a 3 × 3 × 3 supercell with a 19 × 19 × 19 q-mesh. The electron-phonon (e-p) coupling properties are obtained using the Quantum Espresso package^39^ with ultrasoft pseudopotentials, energy cutoff of 40 Ry and a q-grid of 8 × 8 × 8.

## Results and Discussion

**Figure 1:**
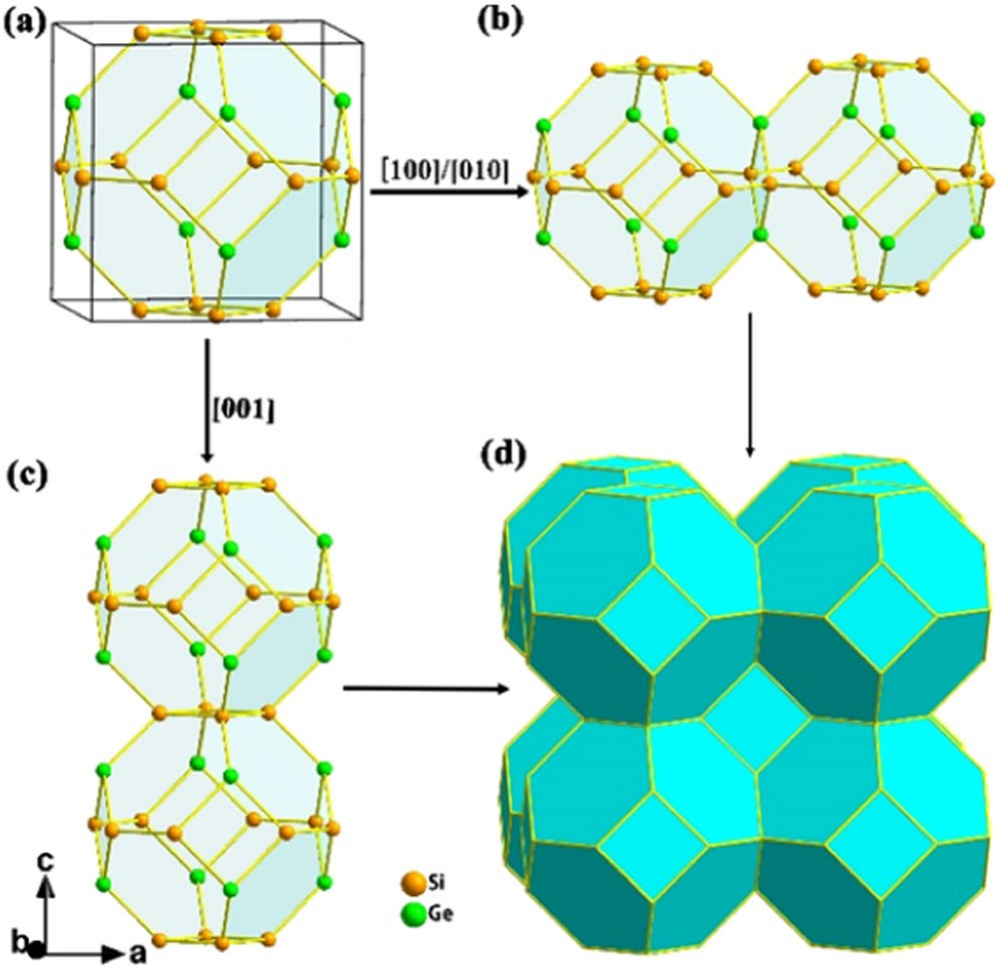
(**a**) The unit cell structure of VII-type Si_2_Ge clathrate marked by black lines (**b,c**) Linkage of tetrakaidecahedra along selected directions. (**d**) The 3D sodalite framework of Si_2_Ge. Tetrakaidecahedron (Si_8_Ge_4_)2: blue polyhedron, yellow apex: Si atom; green apex: Ge atom.

The stable structure of Si_2_Ge obtained from global structure search is shown in Fig. 1. The optimized Si_2_Ge crystallizes in the Tetragonal space group, *I*4/mmm (no. 139), with a = b = 6.759 Å, c = 6.868 Å (Fig. 1). The lattice strain is mostly induced by the distorted tetrahedral coordination of SiGe alloy, or, alternatively, by the 90.2° (∠GeSiGe) and 89.7° (∠SiGeSi) of 4-membered (Si_2_Ge_2_) rings along c direction. The 3D framework is composed of a 24-atom tetrakaidecahedra (Si_8_Ge_4_)2 formed by four-fold coordination of Si at 8*j* and Ge at 4*d* sites (Fig. 1b,d). The clathrate-forming polyhedron is a truncated octahedron, so-called clathrate-VII pattern^40^, formed by six quadrangles and eight hexagons ([4^6^6^8^]). The Si_2_Ge-VII clathrate is 0.11 and 0.06 eV/atom lower in energy than Si and Ge-VII clathrates, but higher than those well-known Si-II and Si-VIII clathrates (0.10 and 0.07 eV/atom) because of containing a large number of four-membered rings resulting strained in comparison to type II frameworks^41^. The bond lengths in Si_2_Ge clathrate are 2.37 Å for Si-Si and 2.45 Å for Si-Ge, respectively. These values are slightly larger than 2.35 Å for *diamond*-Si, 2.38 Å (Si-Si) for Type-I Si clathrate^42^, 2.36‒2.42 Å (Si-Ge) for Si_34-x_Ge_x_ alloy clathrate^6,18^. Generally, a longer bond length corresponds to weaker bond interactions, and weak bond interaction decrease the speed of the sound, which conversely drop the thermal conductivity of the lattice^43^. Therefore Si_2_Ge-VII clathrate shows relatively stable and weak covalent bonds which is responsible for the low lattice thermal conductivity.

**Figure 2:**
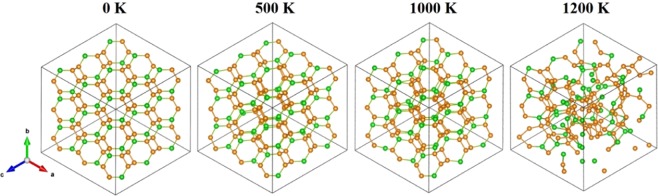
Snapshot of the Si_2_Ge at 10 ps of the ab initio molecular dynamics simulation in the NVT ensemble. The optimized Si_2_Ge was used as the initial structure. The temperature of the system was controlled at 500 K, 1000 K and 1200 K. The estimated melting temperature is around 1200 K. Yellow apex: Si atom; green apex: Ge atom. The dotted red lines represent the broken bonds.

Also, we simulate the thermal stability of Si_2_Ge clathrate. A 3 × 3 × 3 supercell is used in the simulations at temperatures of 500, 1000 and 1200 K by performing ab initio molecular dynamics (AIMD) simulations. The snapshots of the geometries at the end simulations show that Si_2_Ge clathrate can maintain its original configuration at temperature up to 1000 K (Fig. 2). At 1200 K, some bonds begin to break and lead to cage structure distorted. The radical distribution functions (RDF, Fig. S2, Supplementary Information) at 500 K and 1000 K have also shown the typical feature of VII-type clathrate. When the temperature reaches 1200 K, RDF exhibit a few feature of liquid. This indicates that Si_2_Ge has a melting/decomposition temperature close to that of Si and Ge-based clathrates. For instance, Ba_8_Al_15_Si_31_ melts at 1073 K^44^, and Sr_8_Ga_16_Ge_30_ melts congruently at 1033 K^45^. The well-preserved geometry of Si_2_Ge at such high temperature suggests the thermal stability of Si_2_Ge clathrate and its possible utilization at a high temperature.

**Figure 3:**
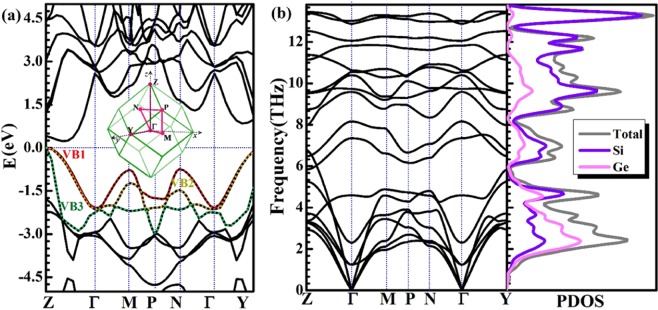
(**a**) Electronic band structure of Si_2_Ge clathrate, the top three valence bands are lighted by green, red and yellow dot lines. Inset: The first Brillouin zone of Si_2_Ge clathrate with high symmetry points (pink points). The fermi level sets 0 eV. (**b**) Calculated phonon dispersions and corresponding phonon density of states.

Figure 3a and Table 1 show the band structure, effective mass and carrier mobility for Si_2_Ge. It is shown that an indirect band gap of 0.23 eV for Si_2_Ge from Fig. 3a. The valence band maximum (VBM) is located at the Z point with 3-degeneracy, are named by VB1, VB2 and VB3 in Fig. 3a. The conduction band minimum (CBM) is along the Z-Γ line of 2-degeneracy imposed by the symmetry of the Brillouin zone which is shown in the inset of Fig. 3a. It is obvious that p-type doped will display slightly higher degeneracy of carrier pockets than that of n-type doped Si_2_Ge. It is well known the Seebeck coefficient is proportional to the density of state effective mass^2,46^, given by *m*_d_^*^ = *N*_v_^2/3^*m*_s_, where *N*_v_ represent the number of degenerate. *m*_s_ can be obtained by *m*_s_ = (*m*_1_*m*_2_*m*_3_)^1/3^. Accordingly, *m*_d_^*^ of valence band is 1.30 and 0.64 m_0_ for VB1(2) and VB3 receptively, while 0.57 m_0_ of conduction band, indicating heavier hole mass behavior.

**Table 1:** Band properties around the Fermi level. *h* and *l* refer to the heavy-mass band light-mass band.

		*m*_1_ (m_0_)	*m*_2_ (m_0_)	*m*_3_ (m_0_)	*m*_s_ (m_0_)	*m*_I_^*^ (m_0_)	*m*_d_^*^ (m_0_)	*µ* (cm^2^/Vs)	*τ* (fs)	*N*_v_	*E*_1_ (eV)
hole	VB1,2 (*h*)	2.26	1.27	0.19	0.82	0.46	1.30	19	11	2	7.44
	VB3 (*l*)	0.47	0.32	0.44	0.40	0.40	0.64	53	30	1	
electron		0.27	0.27	1.22	0.45	0.36	0.57	83	47	2	6.54

effective mass along three principle directions (*m*_1/2/3_) calculated by EMC package^66^, m_0_ is the free electron mass), the density of states effective mass of a single band (*m*_s_), inertial effective mass (*m*_I_^*^), density of state effective mass (*m*_d_^*^), carrier mobility (*μ*) and predicted relaxation time (*τ*) for hole and electron of Si_2_Ge at room temperature (300 K), Number of degenerate carrier pocket (*N*_v_), deformation potential constant (*E*_1_).

The optimal ZT performance is determined by the weighted mobility, ZT ∝ *µ*(*m*_d_^*^/*m*_0_)^3/2^ (refs. ^3,46–49^). Taking the assumption of acoustic or optical phonon scattering are predominant for charge carriers, the mobility can be expressed as *µ*∝1/(*m*_s_^3/2^*m*_I_^*^), as mentioned in (1). Additionally, the optimal ZT ∝*N*_v_/*m*_I_^*^, is inversely proportional to *m*_I_^*^ (ref. ^2^). *m*_I_^*^ can be calculated by *m*_I_^*^ = 3/(1/*m*_1_ + 1/*m*_2_ + 1/*m*_3_). The mobility of n-type Si_2_Ge can be estimated to be 83 cm^2^/Vs using the average *m*_I_^*^ = 0.36 m_0_ of conduction band. Then, we can estimate the constant carrier scattering time *τ* = 47 fs at 300 K for n-type Si_2_Ge. Similarly, the hole mobility and its relaxation time are also listed in Table 1. Consequently, the multiple degenerate valence band (VB1 and VB2) producing a large *m*_d_^*^ and thereby a high *S* with explicitly reduced the hole mobility. Compared with the valence band, the light *m*_d_^*^ and *m*_I_^*^ of the conduction band is beneficial to increase *µ* and then enhance ZT performance. Therefore, it is clear that the light mass plays a crucial role in carrier transport and TE performance^48^.

Generally, the deformation potential (DP) theory overestimates the mobility due to the neglect of scatterings from other phonon modes^49^. The calculated average e-p coupling constant (λ) is to be about 0.082 from the dominated three acoustic branches using Quantum Espresso package. Such weak e-p coupling indicates that the low carrier scattering rates from e-p coupling and large carrier relaxation time of e-p coupling. The detail e-p coupling constants vs. frequency is shown in Fig. S3 (Supplementary Information). Seen from Fig. S3, low frequency phonons, especially those less than 2 THz, have greater e-p coupling than that of high frequency phonons and have strong carrier scattering rates. The phonons in this region are mainly derived from the acoustic branches. Therefore, for Si_2_Ge, deformation potential method can give a reasonable carrier relaxation time.

**Figure 4:**
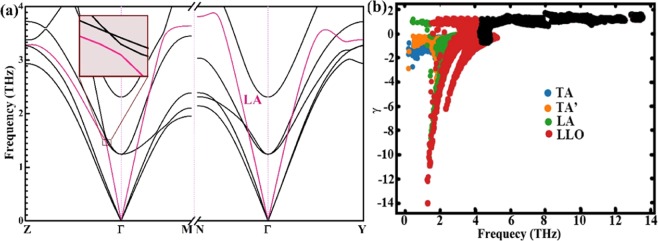
(**a**) Details of the low-energy (<4 THz) of the phonon spectrum of Si_2_Ge along the Z–Γ, Γ–M, N–Γ and Γ–Y directions. The green ellipse highlight avoided-crossing points between the acoustic and optical modes. (**b**) Grüneisen parameter for Si_2_Ge.

Fig. 3b shows the calculated phonon structure of Si_2_Ge clathrate. The low frequency vibrations, <4 THz, are strongly contributed from Ge atoms. Three extremely anomalous low-lying optical (LLO) phonons are overlapped with the longitudinal acoustic (LA) phonons. The boundary frequency of LLO1 branch at the Γ point is about 1.2 THz (43 cm^−1^), is similar to most of the LLO phonons in other low *κ*_L_ PGEC compounds, for example, Yb filled skutterudites (42 cm^−1^)^50^ and Ba_8_Ga_16_Ge_30_ (44 cm^−1^)^51^. LLO branches have such large phonon dispersion slope near the Γ point, which means high phonon velocity and strong anharmonic behaviour and may be provided essential scattering channels for heat-carrying phonons, similar to that of PbTe^52–54^. More importantly, the “avoided crossing” interaction between LLO and longitudinal acoustic (LA) branches has been observed in Fig. 4a along Z-Γ line at 1.5 THz. There is a small gap at avoided crossing point indicates strength of coupling between LA and LLO modes seen from the inset of Fig. 4a. It leads to enhance the phonon scattering rates and reduce acoustic mode velocities, and then result the low *κ*_L_.

Figure 4b shows the Grüneisen parameter (γ) for Si_2_Ge as a function of the phonon frequency. The γ shows similar features as the Si-VII^55^, where negative γ are spread out at low frequency values. TA and LLO branches possess high absolute γ, typically, the minimum γ is extraordinarily low ~−14.16. The average Grüneisen parameter calculated from ShengBTE is 3.19 at 300 K. This value is a little larger than that of AgSbSe_2_ (3.05, a low thermal conductivity material, 0.48 W/mK at 300 K)^56^. The acoustic and LLO modes have much larger absolute γ and play an important role in lattice thermal resistance of Si_2_Ge.

**Figure 5:**
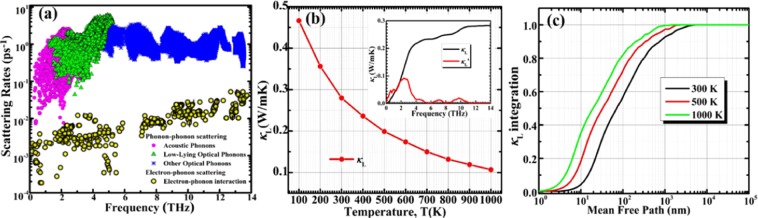
(**a**) Phonon-phonon scattering rates vs. frequency of acoustic (pink circles), low-lying optical (green triangles), other optical (blue stars) phonons and electron-phonon interaction scattering rates (yellow circles) for Si_2_Ge calculated at 300 K. (**b**) Lattice thermal conductivity *κ*_L_ as a function of temperature for Si_2_Ge. The inset shows accumulated lattice thermal conductivities with respect to frequency (black lines), and the derivatives (red lines). (**c**) Normalized *κ*_L_ integration for Si_2_Ge with respect to the phonon MFP at 300, 500 and 1000 K.

The phonon scattering rates (SC) related to phonon-phonon interactions (PPI) and electron-phonon (EPI) are shown in Fig. 5a. The phonon-phonon SC from acoustic phonons is as low as the order of 0.006 ps^−1^, while the low lying optical phonons is in the range of 0.06~8 ps^−1^ and are 1–2 orders of magnitude higher than acoustic branches with frequencies above ~5 THz for Si_2_Ge clathrate. High SC around 5 THz from flat optical phonons. One can see the electron-phonon SC due to EPI is much smaller than the phonon-phonon scattering. Si_2_Ge has stronger lattice anharmonicity, as a consequence, electron-phonon scattering nearly has no contributions to the lattice thermal transport.

Based on ShengBTE, Si_2_Ge actually possess a low lattice thermal conductivity seen from Fig. 5b. With the temperature rising the lattice thermal conductivity decreases monotonically. At 300 K, lattice thermal conductivity is 0.28 W/mK, which is lower than majority of clathrates, such as Sr_8_Ga_16_Ge_30_ (0.9 W/mK)^45^, Sn-based clathrates (~1 W/mK)^57^, and comparable to the unconventional transition metal-phosphorus clathrates with ordered superstructures and heavy elements, such as Ba_8_Cu_16_P_30_ (~0.3 W/mK)^58^ and Ba_8_Au_16_P_30_ (~0.2 W/mK)^59^. At 1000 K the lattice thermal conductivity decreases dramatically to ~0.12 W/mK, which is lower than that measured for SnSe single crystals at 973 K (0.23 ± 0.03 W/mK)^60^. The inset of Fig. 5b shows the cumulative lattice thermal conductivity vs. phonon frequnency of Si_2_Ge. We found that the lattice thermal conductivity increases quickly with *ω* in the low-frequency region. By setting a cutoff of 4 THz, the accumulated thermal conductivity is found to be as high as ~73%, which means low frequency (<4 THz) phonons may make an importance role on *κ*_L_ due to low scattering rates because of large group velocity of acoustic modes which are mainly from vibration of Ge discussed in the previous description (see Fig. 3b). The high-frequency optical phonons have SC of 1 ps^−1^, which are less contribution on heat current. The cumulative lattice thermal conductivity divided by total lattice thermal conductivity of Si_2_Ge with respect to phonon mean free path (MFP) at 300, 500 and 1000 K, are plotted in in Fig. 5c. As the MFP increases, the normalized *κ*_L_ integration increases, and then approaches 1. It is found that the thermal conductivities are dominated by phonons with MFPs ranging from 0.1 to 5 µm at room temperature. At width about 70 nm, the lattice thermal conductivity drops about 50%. At high temperatures, the phonon MFPs become even shorter, the MFP corresponding to the median *κ*_L_ accumulation in Si_2_Ge reduces from 33 nm at 500 K to 19 nm at 1000 K. The phonon MFPs in Si_2_Ge are notably longer than those in other clathrate (around 10 nm at 300 K for Type-I Si clathrate)^61–63^, which means *κ*_L_ of Si_2_Ge is more sensitive to size effects.

**Figure 6:**
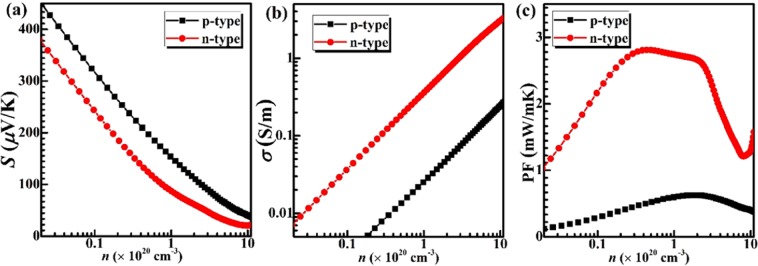
(**a**) Seebeck coefficient *S*, (**b**) Electronic conductivity *σ*, and (**c**) Power factor *S*^2^*σ* (PF) of Si_2_Ge clathrate as a function of carrier concentration at 300 K. n-type (red circle), p-type (black square).

The electronic thermal conductivity (*κ*_e_) was evaluated via Wiedemann-Franz law: *κ*_e_ = *L*_0_*σ*T with *L*_0_ = 2.44 × 10^−8^ W·Ω/K^2^. The Seebeck coefficient *S*, electrical conductivity *σ*, and TE power factor *S*^2^*σ* (PF) as a function of carrier concentration at 300 K have been shown in Fig. 6. Clearly, p-doped Si_2_Ge has the higher Seebeck coefficient than n-dope ones over the full carrier concertation range (0.01~10 × 10^20^ cm^−3^), while the higher conductivity values of electrons than that of holes. This consistent with the discussed above. Since *S* decreases as carrier concentration where *σ* increase, the maximum power factor is 0.63 mW/mK^2^ at the hole concentration of 1.91 × 10^20^ cm^−3^, while 2.81 mW/mK^2^ at the electron concentration of 4.31 × 10^19^ cm^−3^. From Fig. 6c, at 300 K, the n-type power factor is much higher than p-type, which further confirmed that the low effective mass contributes to the enhancement of the TE performance.

**Figure 7:**
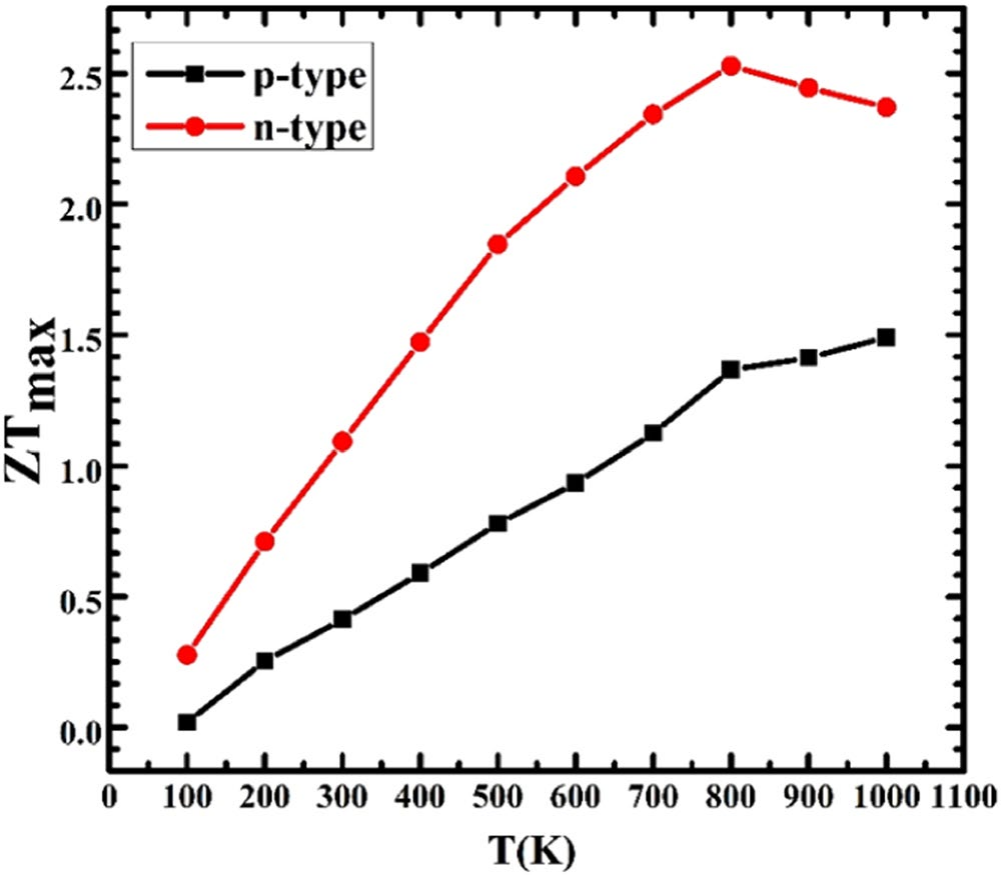
The maximum ZT of Si_2_Ge clathrate as a function of temperature. p-type (black square, holes), n-type (red circle, electrons).

ZT at different temperature vs. carrier concentration is plotted in Fig. S4 (see Supplementary Information). The ZT value is peaked at a specific carrier concentration at the different temperature. For electrons at room temperature, the peaked ZT value is predicted to be 0.41 at 5.41 × 10^18^ cm^−3^ and that for holes is 1.09 at 5.41 × 10^19^ cm^−3^. This peaked ZT value is named the maximum ZT (ZT_max_). ZT_max_ as a function of temperature is plotted in Fig. 7, which demonstrates a linear increase below 800 K and then decrease for n-doped, while a linear increase with temperature for p-doped. The highest ZT_max_ achieved at 800 K is 2.54 for n-doped Si_2_Ge clathrate and 1.49 for p-doped at 1000 K. These values are superior those realized in K_8_Ba_16_Ga_40_Sn_96_ (n-type, 1.12 at 637 K)^64^, and type-I Ba_8_Ga_16_Ge_30_, (p-type, 1.10 at 823 K)^65^.

To summarize, we extend a new clathrate materials, namely Si_2_Ge-VII clathrate on basis of global structure search and density functional theory. This clathrate has a tetrakaidecahedral lattice similar to sodalite and exhibits excellent thermal and dynamical stabilities. Si_2_Ge clathrate has an indirect band gap of 0.23 eV, with higher p-doping Seebeck coefficient owing to higher hole density-of-sates mass and higher n-doping electrical conductivity thanks to lower electron effective mass. Interestingly, it owns a low lattice thermal conductivity due to its weak bonding interaction and strong anharmonic LA-LLO coupling results in avoided-crossing. The fascinating electronic properties together with the low lattice thermal conductivity make Si_2_Ge clathrate a promising TE material. We attribute the remarkably high ZT peak of Si_2_Ge (n-type 2.54 at 800 K and p-type 1.49 at 1000 K). This study would enrich the diversity and boost the development of TE materials.

## Supplementary information

supplementary information.

## Data Availability

The data that support the findings of this study are available from the corresponding authors upon reasonable request.
