# Supplementary material for: Si2Ge: A New VII-Type Clathrate with Ultralow Thermal Conductivity and High Thermoelectric Property
Source: Sci Rep. 2020 Feb 20;10:3068. doi: 10.1038/s41598-020-59820-8 (PMC7033159; doi:10.1038/s41598-020-59820-8)
Supplement: Supplementary file 1 — supplementary information. [file 41598_2020_59820_MOESM1_ESM.docx]

Si_2_Ge: A New VII-Type Clathrate with Ultralow Thermal Conductivity and High Thermoelectric Property

Jinni Shen,^‡ a,b^ Tianzhu Xie, ^‡ a^ Longkun Zhang,^a^ Ping Wang,^a^ Zhenxing Fang,^*c^

a. College of Materials Science and Engineering, Fuzhou University, Fuzhou, 350108, China

b. Key Laboratory of Eco-materials Advanced Technology, College of Materials Science and Engineering, Fuzhou University, Fuzhou 350108, China

c. Department of Physics, Zunyi Normal University, Zunyi, Guizhou, 563006, China

**DEFORMATION ENERGY**

Deformation energy defined by $E_{1}^{i}=\Delta E_{i}/(\Delta V/V_{0})$. Here Δ*E_i_* is the energy change of the *i*th band under proper cell compression and dilatation (calculated using a step of 0.3%), *V*_0_ is the volume of lattice and *ΔV* is the deformation of *V*_0_. All structural properties in the calculation of relaxation time were obtained from DFT and properties related to the electronic structure were computed with the HSE06 functional. In this paper, the absolute CBMs and VBMs of Si_2_Ge are computed by applying methodology for referencing bulk electronic levels to the vacuum that involves both bulk calculations and the surface/slab calculations. This approach has been employed previously for computing work functions of metals and ionization potentials of semiconductors.


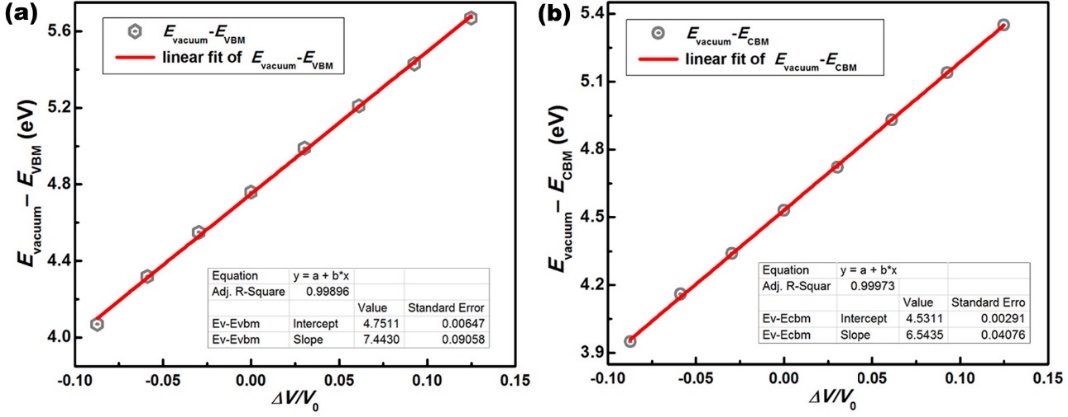


**Fig. S1**. Band energy of the VBM (a) and CBM (b) of Si_2_Ge clathrate with respect to the vacuum energy as a function of lattice dilation. Band energies were calculated with the HSE06 functional. Red solid lines are the fitting curves. Insets show the standard errors of the fitted slope, which corresponds to the deformation potential.


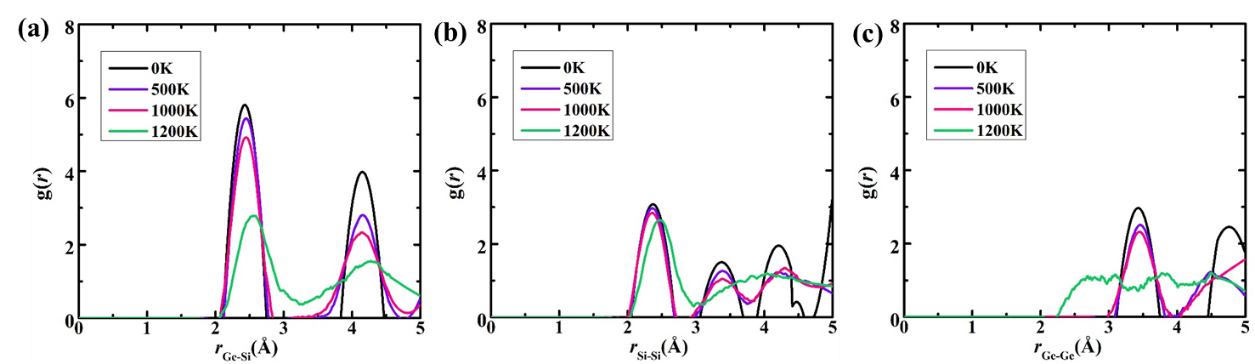


**Fig. S2.** Radial distribution functions (RDFs) of Si_2_Ge at temperatures spanning from 500 to 1200 K.


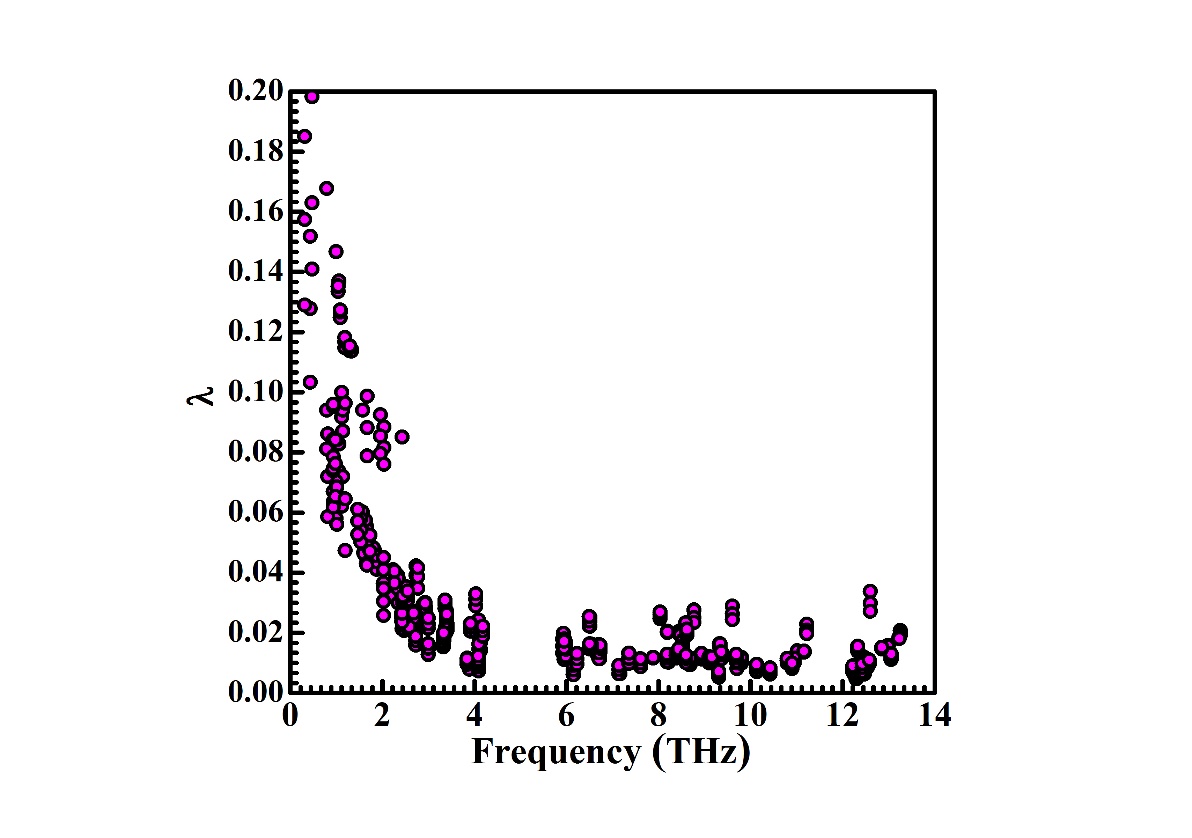


**Fig S3.** e-p coupling constants (λ) vs. frequency.


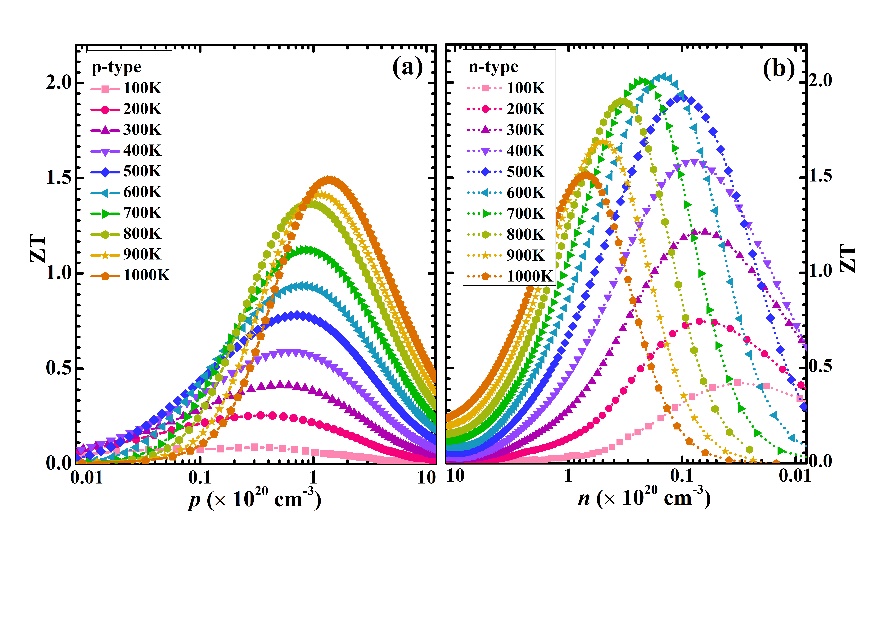


**Fig. S4.** Thermoelectric figure of merit ZT of Si_2_Ge as a function of carrier concentration with different temperature arrange from 100 to 1000 K. (a) p-type (solid lines, holes) (b) n-type (dot lines, electrons)

**Table S1.** Average longitudinal (LA) and transverse (TA/TA') Debye temperatures (Θ_TA/TA'/LA_), and phonon velocities (*v*_TA/TA'/LA_) in Si_2_Ge calculated from the phonon dispersion (Fig. 3b), and corresponding agave values are also listed in the last column. The Debye temperature is calculated using Θ = *ω*_D_/*k*_B_ (*ω*_D_ is the largest acoustic frequency in each direction); the phonon velocity is the slope of the acoustic phonon dispersion around the point. The grüneisen parameters, Debye temperatures, and phonon velocities are averaged by the weight of the high-symmetry points.

|  | TA | TA' | LA | Agv. |
| --- | --- | --- | --- | --- |
| Θ(K) | 144 | 158 | 158 | 154 |
| υ(m/s) | 2542 | 2863 | 5728 | 3020 |
